# Clinical leadership and hospital performance: assessing the evidence base

**DOI:** 10.1186/s12913-016-1395-5

**Published:** 2016-05-24

**Authors:** F. Sarto, G. Veronesi

**Affiliations:** Department of Economics, Management, Institutions, “Federico II” University of Naples, Naples, Italy; Accounting and Finance Division, Leeds University Business School, Leeds, UK

**Keywords:** Clinical leadership, Hospital performance, Narrative review

## Abstract

**Background:**

A widespread assumption across health systems suggests that greater clinicians’ involvement in governance and management roles would have wider benefits for the efficiency and effectiveness of healthcare organisations. However, despite growing interest around the topic, it is still poorly understood how managers with a clinical background might specifically affect healthcare performance outcomes. The purpose of this review is, therefore, to map out and critically appraise quantitatively-oriented studies investigating this phenomenon within the acute hospital sector.

**Methods:**

The review has focused on scientific papers published in English in international journals and conference proceedings. The articles have been extracted through a Boolean search strategy from ISI Web of Science citation and search source. No time constraints were imposed. A manual search by keywords and citation tracking was also conducted concentrating on highly ranked public sector governance and management journals. Nineteen papers were identified as a match for the research criteria and, subsequently, were classified on the basis of six items. Finally, a thematic mapping has been carried out leading to identify three main research sub-streams on the basis of the types of performance outcomes investigated.

**Results and contribution:**

The analysis of the extant literature has revealed that research focusing on clinicians’ involvement in leadership positions has explored its implications for the management of financial resources, the quality of care offered and the social performance of service providers. In general terms, the findings show a positive impact of clinical leadership on different types of outcome measures, with only a handful of studies highlighting a negative impact on financial and social performance. Therefore, this review lends support to the prevalent move across health systems towards increasing the presence of clinicians in leadership positions in healthcare organisations. Furthermore, we present an explanatory model summarising the reasons offered in the reviewed studies to justify the findings and provide suggestions for future research.

## Background

Over the last three decades healthcare organisations (in particular hospitals) have progressively adopted models of governance alternative to the traditional ‘professional bureaucracy’ [1], which are influenced by the managerialist ideology [2]. In the public sector, these changes have signified a ‘corporatisation’ of hospitals towards a business-like form [3], in the aim to extend the independence of decision making’ [4]. This shift has implied the introduction of significant structural changes in leadership positions, namely the creation of the Chief Executive Officer (CEO) role and the appointment of boards of directors [5]. Following the fundamental underlying assumption in corporate governance that better governance mechanisms lead to greater efficiency and effectiveness of organisations, the adoption of these business-like governance arrangements has been seen as crucial for performance improvements in private (profit and non-profit) and public hospitals.

While these reforms have been largely supported in relation to increased organisational autonomy and greater independence in strategic decision making processes [6], what is the most effective bundle of expertise and skills of hospital senior leaders has remained an open issue. In this sense, scholars and practitioners have questioned whether the hospital CEO and the board directors should be clinically-trained top managers [7–10]. Empirical evidence suggests that the extent to which clinicians are involved in leadership positions varies greatly across countries. For instance, analysing a sample of 6,500 US hospitals Gunderman and Kanter [11] report that only 235 organisations were led by doctors. Similar conclusions are drawn in the UK, where Veronesi et al. [12] suggest that on average individuals with a clinical background make up over a quarter of the board members (26.03 %) and represent about 22 % of the hospital CEOs. Markedly different conclusions are, on the other hand, reported in relation to healthcare organisations in continental Europe. Indeed, comparative research highlights how doctors represent the majority of senior managers in most European hospital systems such as Italy (50 %), France (63 %) and Germany (71 %) [13].

What the available research has in the main suggested is that greater clinicians involvement in leadership positions, especially at the strategic level, has potentially important benefits for healthcare outcomes [12, 14–17]. Accordingly, it has been pointed out that ‘in a healthcare system that is complex, troubled, and challenging, the doctor CEOs and board directors bring a unique set of skills to the business of medicine’ [10], as they better understand clinical challenges and general patients’ needs [18, 19]. Additionally, they can ensure better communication with clinically-qualified personnel as well as enjoying greater legitimacy [13, 20]. Thus, through greater involvement of clinicians at the strategic level, hospitals not only will benefit from a higher quality of strategic decisions, but also from a more concrete implementation of decisions taken.

Yet, although the evidence shown in these studies seems supportive, questions remain about the impact of clinical leadership on overall hospital performance. For instance, concerning the consequences of clinical involvement for the quality of the service provided, while Goodall [7] reports a significant relationship between clinical CEOs and organisational performance in the US, Veronesi et al. [12] do not observe any significant association between the clinical background of the CEO and service quality. The apparent inconclusiveness of the extant research is even more obvious if we consider the impact of clinical leadership on hospital financial performance. On the one hand, a number of studies show that clinically-qualified managers positively affect hospital financial efficiency [14]. Conversely, other studies in the US and Italy highlight a negative impact of clinical leadership on the efficient management of hospital resources [21, 22].

Thus, while the possibility that clinicians’ involvement could be beneficial for hospital has been recognized [13], to what extent managers with a clinical background might specifically affect different types of hospital performance outcomes is still poorly understood. Indeed, to our knowledge there has been no systematic review of the quantitative evidence investigating this phenomenon. Therefore, the purpose of the paper is to fill this gap by mapping out and critically appraise quantitatively-oriented studies analysing the association between clinicians’ involvement in senior leadership positions (i.e. CEO, top management and board of directors) and hospital performance. This represents a much needed contribution considering that a positive impact on performance outcomes underpins the widespread drive for enhancing clinical leadership in healthcare organisations [23]. Furthermore, the paper builds on the evidence reported by presenting an explanatory model, where reasons for the positive/negative impact of clinical leadership are offered.

The remainder of this paper is structured as follows. Section 2 presents the review method. Section 3 illustrates the findings and the literature systematization. Section 4 concludes by presenting the explanatory model and offering suggestions for future research on this topic.

## Review method

**Fig. 1:**
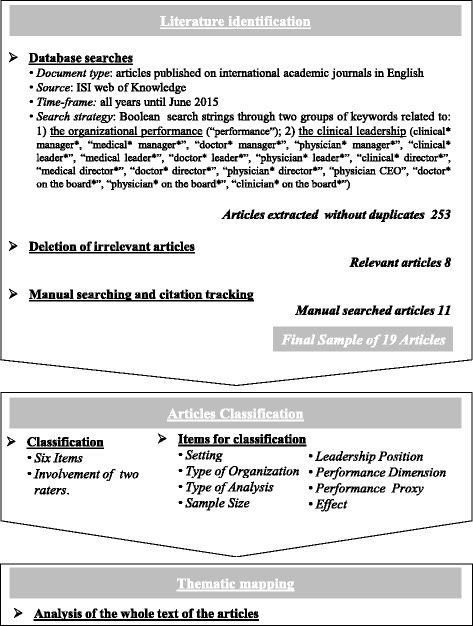
Research Design

Following previous literature [24, 25], our systematic review is based on a narrative approach. This is consistent with the aim of our study of summarizing and interpreting key findings of research exploring the involvement of clinicians in governance roles and its impact on hospital performance, as well as recognizing different narratives of the phenomenon. To identify the relevant articles, we have focused on scientific papers published in English in both peer-reviewed international journals and conference proceedings. This is a standard practice in literature reviews since these sources are considered ‘certified knowledge’ which enhances the reliability of the analysis [26]. We have not imposed time constraints on the search process to be able to capture all relevant contributions until June 2015 (Fig. 1).

In order to extract relevant papers, we have conducted Boolean searches using truncated combinations of two groups of search strings [27]. While one string covers the performance realm, the other one includes keywords referred to clinical leadership. We applied these criteria to the ISI Web of Science citation and search source. We searched for all papers whose title, publication, abstract and keywords included at least a string belonging to one of the two selected sets. We have opted for this database because it is considered the largest, most commonly used and generally accepted source for literature reviews [26, 27]. After removing potentially duplicated articles, the search returned 253 papers. To assess the relevance and consistency of these studies with the selection criteria, each of them was evaluated in relation to title, keywords, abstract and, when necessary, full text. This selection process was carried out by the two authors only focusing on quantitative empirical studies. We decided to not consider qualitatively-oriented articles as their findings are not generalizable. Eventually, we excluded 245 not relevant articles. Furthermore, following the process adopted in previous literature reviews [28], our search was completed by manually looking and tracking citations for additional relevant articles in highly ranked healthcare management journals. Therefore, the final sample consists of 19 papers (8 identified through database search and 11 from the manual search and citation tracking phase).

Following a standard approach in systematic literature reviews [26, 27], we have analysed and classified the selected papers according to the following criteria: research settings, type of organisation, type of analysis, sample size, leadership position, performance dimension, performance proxy, and effect of clinical leadership on organisational performance. Finally, in order to better interpret the findings of the studies included in the final sample and to fully understand the explanations provided for the reported results, a thematic mapping of the articles was carried out [24]. We iteratively identified the key themes and independently analyzed the articles and discussed their conclusions. This process has allowed us distinguishing three main research sub-streams according to the performance outcomes used as dependent variable(s): (i) management of financial and operational resources, (ii) quality of the care provided and (iii) social performance of the hospital. More specifically, while articles belonging to the first stream investigate the implication of clinicians’ involvement in leadership positions for financial performance, the second and the third ones mainly explore the effect on non-financial performance.

## Analysis of the literature

**Fig. 2:**
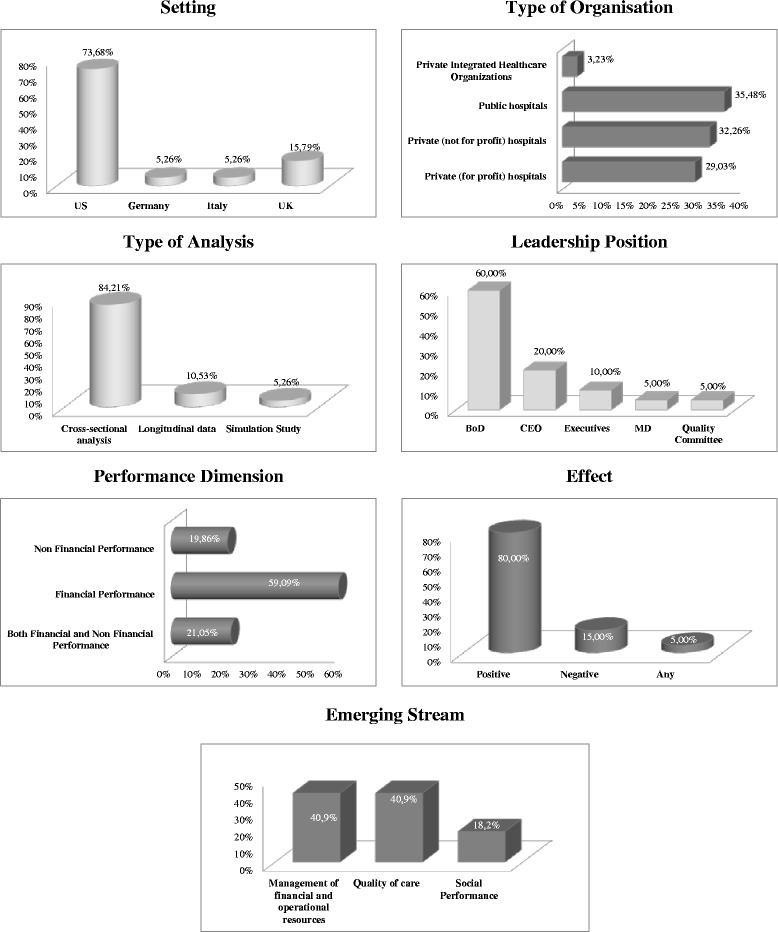
Descriptives

Table 1 provides an overview of the selected articles. These studies have been conducted between 1993 and 2015, with the majority of them (63 %) published in the last decade (2006–2015). The results of the categorisation process show that most of the articles focused on US settings (74 %). Nevertheless, studies based on the German, Italian and British health systems are also represented in the final sample. Concerning the type of organisations investigated, most papers (60 %) focused on private healthcare organisations, in both for-profit and not-for-profit sectors, while relative less attention has been devoted to public sector hospitals (35 %). This can be explained by the fact that the early studies almost exclusive concentrated on the US health system. Regarding the type of analysis conducted, the majority of the studies (84 %) have employed a cross-sectional approach, with a minor percentage adopting longitudinal data analysis (11 %) and simulation techniques (5 %). The average size of the investigated samples stood at around 237 organizations, although there are wide variations between studies (from 2 to 1,220). With reference to leadership positions, the extant literature has mainly investigated the involvement of clinicians in boards of directors (60 %) and in CEO positions (20 %), with a residual percentage focusing on top executives, quality committee and medical director positions. Finally, with regard to the performance dimension, most studies have focused on the impact of clinically-qualified managers on financial or operational performance (59 %), which is not too surprising considering the prevalence of private organisations as research setting. Furthermore, although the majority of the studies analysed only one dimension of organisational performance, some articles have focused on both financial and non-financial (including social) performance outcomes (21 %) (Fig. 2).

**Table 1:** Articles analysis

Study	Setting	Type of organisation	Type of analysis	Sample size	Leadership position	Performance dimension	Performance proxy	Effect	Emerging Stream
Bai [44]	US	Private (for-profit) hospitals	Cross-sectional analysis	137	BoD^a^	Non-financial	Community Benefits (uncompensated care cost, net education expense, and net research expense, scaled by hospital gross patient revenues)	+	Social Performance
Bai and Krishnan [36]	US	Private (not-for-profit) hospitals	Cross-sectional analysis	142	BoD	Non-financial	Quality of Care (process of care quality rating)	+	Social Performance
Brickley et al. [47]	US	Private (not-for-profit) hospitals	Cross-sectional analysis	228	BoD	Financial	Donations	−	Social Performance
De Andrade Costa [45]	US	Private (not-for-profit) hospitals Private (for-profit) hospitals	Longitudinal data analysis	281	BoD	Non-financial	Community Benefits (uncompensated care)	+	Social Performance
Goes and Zhan [29]	US	Private (for-profit) hospitals	Longitudinal data analysis	300	BoD	Financial	Operational Profitability (operating margin) Occupancy (average daily occupancy) Hospital Costs (operating expenses/1000 patient-days)	+	Management of financial and operational resources
Goldstein and Ward [33]	US	Public hospitals Private (for-profit) hospitals Private (not-for-profit) hospitals	Cross-sectional analysis	200	Executives	Non-financial	Operational Efficiency (occupancy rate, market share)	+	Management of financial and operational resources
Goodall et al. [37]	US	Private (for-profit) hospitals Private (not-for-profit) hospitals	Cross-sectional analysis	10	CEO	Non-financial	Reputation with specialists (survey)	+	Quality of care
Goodall [7]	US	Public hospitals Private (for-profit) hospitals Private (not-for-profit) hospitals	Cross-sectional analysis	100	CEO	Non-financial	Index of Hospital Quality in the area of Hospital Structure (availability of resources), Outcomes (mortality rate, patient safety index) and Process (reputation scores based on survey)	+	Quality of care
Jiang et al. [15]	US	Public hospitals Private (for-profit) hospitals Private (not-for-profit) hospitals	Cross-sectional analysis	490	Quality Committee	Non-financial	Quality of Care Process (20 measures covering heart attack, heart failure, pneumonia, and surgical infection prevention) Quality of Care Outcomes (mortality rate in the area of heart attack, heart failure, pneumonia)	+	Quality of care
Kuntz and Scholtes [40]	Germany	Public hospitals	Cross-sectional analysis	604	MD^b^	Non-financial	Clinical Quality (nurses-to-patients ratio and physicians-to-patients ratio).	+	Quality of care
Molinari et al. [30]	US	Public hospitals Private (for-profit) hospitals Private (not-for-profit) hospitals	Cross-sectional analysis	190	BoD	Financial	Profitability (hospital operating margin, net income to patient revenues, return on total asset) Liquidity (days in accounts receivable) Capital Structure (long-term debt to total assets, bad debt and charity) Capital Intensity (net plant, property and equipment per bed; hospital occupancy rate)	+	Management of financial and operational resources
Molinari et al. [31]	US	Private (for-profit) hospitals	Cross-sectional analysis	190	BoD	Financial	Profitability (operating margin)	+	Management of financial and operational resources
Prybil [32]	US	Public hospitals Private (for-profit) hospital	Cross-sectional analysis	14	BoD	Financial & Non-Financial	Quality of Care rating (mortality index, complications index, patient safety index, core measures score, readmission rate, length of stay, case-mix and wage-adjusted inpatient expense per adjusted discharge) Profitability (operating profit margin) Patient Satisfaction (patient ratings)	+	Management of financial and operational resources Quality of care
Sarto et al. [22]	Italy	Public hospitals	Cross-sectional analysis	96	CEO	Financial & Non-Financial	Profitability (operating margin) Financial Efficiency (expenses-to-beds ratio) Quality of care (appropriateness) Efficiency of care (length of stay)	+/−	Management of financial and operational resources Quality of care
Schultz and Pal [38]	US	Private integrated healthcare organisations	Simulation Study	2	CEO	Financial & Non-Financial	Profitability (net income) Quality of Care	nd	Management of financial and operational resources Quality of care
Succi and Alexander [21]	US	Public hospitals Private (for-profit) hospitals Private (not-for-profit) hospitals	Cross-sectional analysis	1,220	BoD Executives	Financial	Operational Efficiency (ratio of total operating expenses divided by adjusted hospital admissions)	−	Management of financial and operational resources
Veronesi et al. [12]	UK	Public hospitals	Cross-sectional analysis	102	BoD	Non-Financial	Quality of care rate (compliance with core standards in the area of health and well-being, clinical effectiveness, safety and patient focus, ease and equity of access)	+	Quality of care
Veronesi et al. [14]	UK	Public hospitals	Cross-sectional analysis	102	BoD	Financial	Financial resources management (quality of the financial resource management rating)	+	Management of financial and operational resources
Veronesi et al. [39]	UK	Public hospitals	Cross-sectional analysis	99	BoD	Non-Financial	Patient Satisfaction Rate (labour access, coordination, information, relationships and comfort)	+	Quality of care

^a^ *BoD* Board of Directors

^b^ *MD* Medical Director

Beyond these stylised facts, in the large majority of the 19 identified papers the effect of clinical involvement in leadership positions on organisational performance was found to be positive. Only three articles showed a negative impact of clinical leadership on organisational outcomes and more specifically on financial performance, and one study highlighted a negative effect on social performance. In the following sub-sections, we present the three sub-streams of the literature that emerged from the thematic mapping of the papers. This analysis suggests that the extant literature has mainly focused on the influence of clinical leadership on the efficient management of resources, the quality of care provided and the social performance of the hospital.

### Clinical leadership and the management of financial and operational resources

Among the studies exploring the impact of clinical leadership on organisational performance, the earliest research sub-stream has focused on its implications for the efficient management of financial and operational resources. These performance outcomes have been typically associated with measures of profitability (such as operating margin and overall profit generated), operational efficiency (bed occupancy rate) and, in few cases, market power (market share). This line of research was firstly developed in the US, where a notable proportion of the acute care provision was (and still is) delivered in private hospitals. However, more recently, this attention to bottom line profitability and competitiveness has also emerged in Europe, in particular as a consequence of the new public management reforms. These reforms have indeed given prominence to efficiency improvements in public sector hospitals in order to make them more business-like [4]. The findings of the studies classified into this sub-stream are not unanimous.

Thus, Goes and Zhan [29] empirically show that greater doctor involvement in the governance of US private acute care hospitals leads to higher bed occupancy and operating margins. Similar conclusions are reported by Molinari et al. [30], who highlight how greater medical staff representation on the board of directors is significantly associated with improved hospital financial performance in terms of profitability, liquidity as well as capital structure and intensity. In a later study, the same authors reinforce these findings by showing that boards with clinicians have significantly better financial performance than boards without any doctor participation [31]. Also focusing on US settings, Prybil [32] finds that non-profit general hospitals outperform the private sector counterparts in terms of overall profitability when doctor involvement in governance roles is higher.

Other studies have investigated the effect of clinician involvement in governance roles for hospital operational efficiency. Accordingly, Goldstein and Ward [33] empirically test the implications of the involvement of doctors in top management positions. Their results support the hypothesis of improved hospital performance in terms of operational efficiency measured as beds occupancy rate and market share. Similar results are reported by Veronesi et al. [14] in a study focused on the English National Health Service (NHS). More specifically, the authors highlight a positive impact of clinical participation on boards of directors of acute care hospital trusts on the financial management of their resources.

Several explanations are offered to motivate the benefits of clinical leadership for the management of financial and operational resources. On the one hand, it is said that clinician involvement in hospital management has been partially driven by pragmatic concerns associated with pressures to control expenditure [12]. Accordingly, Succi and Alexander [21] state that the involvement of doctors in top management positions is frequently sought to reinforce their ‘commitment to cost containment’; that is, doctors turned managers would be influenced in their attitudes and behaviours, and would consequently facilitate the ‘adoption of more cost efficient clinical practices’. There is, indeed, evidence suggesting that clinical leadership can positively stimulate the behaviour of front line staff. For instance, Dorgan et al. [20] posit that managers with a clinical background have more credibility among clinical staff and thus they are more likely to influence the behaviour of their colleagues than non-medical managers. Similarly, it has been observed that in the presence of clinical leadership front-line staff will be less likely against adopting cost-efficient clinical practices as there will be less concerns that the proposed changes will negatively influence the quality of the service provided [14].

In spite of the growing number of studies supporting the advantages generated by clinical leadership for the management of hospital financial and operational resources, some scholars have found evidence of a negative impact. For instance, Succi and Alexander [21] show that the main effect of doctor involvement in hospital management is not related to greater hospital efficiency, but, on the opposite, to lower efficiency. Same conclusions are drawn by Sarto et al. [22], whose findings indicate that CEOs with a medical background have a negative impact on financial performance, calculated as operating margin and financial efficiency, although the evidence seems to suggest that the impact is lower in the case of medical CEOs with previous managerial expertise.

Substantially, it cannot be excluded that the involvement of clinicians in leadership positions can be also counter-productive. This is also conceptually argued by studies not included in the sample but cited. For instance, Mannion et al. [34] find that in hospitals dominated by ‘pro-professional cultures’, strategic and operational decisions can be oriented towards meeting clinical needs at the expense of financial performance targets. Additionally, it has been noticed that when doctors in governance roles gain awareness of the trade-offs faced by managers between financial concerns and clinical priorities, the differences between management and medical worlds can intensify rather than being solved [21]. Moreover, some authors argue that clinical managers can experience strong role conflict when moving into managerial roles which in turn affects their strategic decision making. In this instance, clinically-qualified managers would prioritise clinical needs at the expense of financial efficiency and cost containment [23].

In line with the findings reported by Sarto et al. [22], an additional line of inquiry has recently suggested that doctors performing managerial roles could be less effective than managers without a clinical background in the financial management of resources when they lack a proper managerial training before taking on the role [14]. Accordingly, Kippist and Fitzgerald [35] recognise that among ‘barriers to the effectiveness of the role of hybrid clinician manager’ it is well recognized ‘the lack of management education and skill’. Similarly, Falcone and Satiani [10] state that a ‘successful physician leader must understand the business of medicine as well as or better than he or she understands the practice of medicine’.

### Clinical leadership and the quality of care provided

A growing number of studies has focused on the impact of clinical leadership on the quality of care provided, measured by process and outcomes indicators. It is important to notice that all the studies (bar one exception) included in this review have reported empirical results that strongly support the positive beneficial effects of the presence of clinicians in leadership roles for the quality of care offered in hospitals.

With reference to the US context, Prybil [32] analyses a sample of 14 non-profit general hospitals and finds that the top high performing hospitals are characterised by a higher involvement of doctors in management than the mid-range performers where the performance indicators are a quality of care rating and a patient satisfaction score. Similarly, Bai and Krishnan [36] report that non-profit hospitals without doctor participation on their boards are more likely to deliver lower quality of care, proxied by the process of care quality rating of the Hospital Quality Alliance. Furthermore, focusing only on hospital board quality committees, Jiang et al. [15] show that having a higher doctor participation on committees strongly improves hospital performance in terms of the care process (measured as quality of care of heart attack, heart failure, pneumonia, and surgical infection prevention) and mortality rates.

Similarly, Goodall [7], focusing on the top 100 US hospitals according to the Index of Hospital Quality ranking of the US News and World Report, finds that having a CEO with a medical background generates greater quality improvements that result in a higher quality ranking for the hospital. Comparable conclusions are also reported by Goodall et al. [37]. In this study, the authors highlight how the US’s top 10 psychiatric hospitals, as ranked by the US News & World Report, are exclusively managed by CEOs with a medical background. Different conclusions are drawn by Schultz and Pal [38], who examine the ability of CEOs of integrated healthcare organisations to make effective strategic decisions. More specifically, the study reveals no significant differences between medically-educated and managerially-educated senior managers in their ability to make strategic decisions that maximize the quality of care provided by the organisation.

Turning our attention to the European settings, Veronesi et al. [12] report that a greater ratio of clinical members on governing boards of English hospital trusts generates better ratings of the quality of the service provided, as well as being associated with a reduction in morbidity rates. In a later study, Veronesi et al. [39] also show that the involvement of clinicians on the board of directors improves the overall patient experience of the care provided by acute hospitals when clinical managers operate in a more autonomous organisational form. A similar methodology was also applied to the Italian NHS, where Sarto and colleagues [22] find a positive correlation between the clinical background of public hospital CEOs and quality outcomes, measured as medical and surgical appropriateness as well as patient length of stay in hospital. Finally, focusing on a sample of German hospitals, Kuntz and Scholtes [40] suggest the existence of a positive relationship between a full-time or heavily involved part-time medical director and a higher staff-to-patient ratio, a common measure of quality and safety of the healthcare process.

By deepening also studies not included in the sample, the positive outcomes of the presence of clinically-qualified managers for the quality of the service provided are explained in a variety of ways. First, it is suggested that the involvement of clinicians in strategic decision making represent the most effective option to achieve the critical link between strategic planning and its implementation at the clinical level [33]. Accordingly, Llewellyn [41] notes that managers with a clinical background are in a privileged position to bridge the historical divide between the worlds of cure, care and administration. Essentially, the assumption is that doctors can more easily acquire management expertise while a non-clinical manager would find more difficult to gain sufficiently appropriate medical knowledge. In this sense, some authors state that the involvement of doctors provides unique advantages to the organisational strategic decision making process due to their crucial knowledge as well as their exclusive relationship with patients [18, 19]. Thus, according to Ford-Eichkoff et al. [42], increasing the number of board members with clinical background provides governing boards with a greater breadth of expertise. Similarly, Dorgan et al. [13] claim that clinician involvement in hospital governance leads to an improvement in managerial decision making quality, as they can better understand clinical challenges and general patient needs and they can more effectively communicate with clinical staff. Additional studies suggest that high performing medical top management teams tend to be those with more ‘quality-centred cultures’ [43]. Finally, other authors emphasise how managers with clinical background will not only improve the quality of hospital decision making, but they will be seen as credible leaders and hence will be more likely to attract talented medical personnel [10].

### Clinical leadership and the hospital social performance

A third sub-stream of studies that emerged from the analysis focuses on the impact of clinical leadership on hospital social performance. These articles are generally more recent and only focus on private healthcare organisations, being all based in the US.

Thus, Bai [44] shows that the occupational background of board members influences the hospital social performance. More specifically, the author finds that in for-profit hospitals the presence of doctors on governing boards is positively correlated with hospital social performance, which is measured as a multi-dimensional construct capturing hospital expenditure on community benefits. Similar results are reported in a study by De Andrade Costa [45], also focused on the private hospital sector. The evidence highlights how increased medical membership of boards positively relates to higher levels of uncompensated care provision, thus improving the benefits for the community. The author justifies these results by stating that the ethical beliefs and professional norms of doctors enhance the probability of serving the best interest of patients even in the absence of financial benefits. Considering all the above, it can be concluded that clinical involvement in top management positions increases the pressure on the governing board to enhance the provision of uncompensated care and, as a consequence, boosts overall hospital social performance [46].

Nevertheless, opposite conclusions are reported by Brickley et al. [47], who investigate the potential conflict of interest in non-profit hospitals between donors and doctors. In this instance, a negative relationship between doctor representation on the governing board and the amount of private donations is found. The explanation provided for this finding is that donors would be deterred to provide resources to the hospital due to the risk that these were subsequently expropriated by doctors. Accordingly, in some studies [48] it is suggested that private doctors are more likely to use hospital resources to maximize their own income rather than directing the funding received towards the types of investment preferred by donors, normally oriented towards community benefits at large. The risk of potential expropriation of hospital resources leads to a reduction in the amount private donations and, hence, a lower likelihood of positive social performance for private hospitals [49].

## Conclusions

Following the growing interest of policy makers and practitioners in the involvement of clinicians in the governance and management of healthcare organisations [9, 50], research conducted on this topic has suggested that greater clinician participation at the strategic decision making level potentially has a wide range of benefits for hospitals. This systematised review of the literature highlights how quantitative analyses of this phenomenon have developed along three main sub-streams. More specifically, the extant research has explored the implications of clinical leadership for the management of financial and operational resources, the quality of care provided and hospital social performance. Although based on a relatively limited number of studies, our analysis overwhelmingly supports the assumption that greater clinical presence is beneficial for hospital decision making processes, which clearly has important implications for policy and practice. Fundamentally, clinical leadership has been found to enhance efficiency and effectiveness of hospitals along a number of performance indicators [12, 14–17]. However, while there is a growing body of evidence to support this conclusion, there are also reasons to question the assumption that the influence will necessarily be positive.

**Fig. 3:**
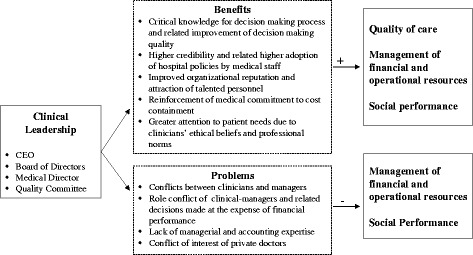
The explanatory model

To further understand the results of this review, in Fig. 3 we present an explanatory model that offers a brief recap of the evidence-base as well as the motivations provided for the positive and negative impact of clinical leadership on hospital performance.

As shown in the figure, it is suggested that the beneficial impact of greater clinician involvement has been justified by the following reasons: (i) greater provision of critical knowledge for the decision making process and consequent improvement of the overall decision making quality; (ii) higher credibility of clinical leaders and related higher adoption of hospital policies by medical staff; (iii) improved organisational credibility and reputation and therefore higher likelihood of attraction of talented personnel; (iv) reinforcement of medical commitment to cost containment; (v) greater attention to patients needs due to ethical beliefs and professional norms of clinicians. Conversely, the negative implications for the management of financial resources and hospital social performance have been linked to the following motives: (i) emergence of conflicts between clinicians-turned managers and career managers, (ii) rise of role conflicts in managers with a clinical background and consequent decisions made at the expense of financial performance to protect clinical needs; (iii) lack of managerial and accounting expertise of clinically-qualified managers; and (iv) conflicts of interest of private doctors in leadership positions.

Despite the evidence overwhelmingly pointing towards a positive impact of clinical leadership on hospital performance, there is clearly scope to further investigate the phenomenon. First, the large majority of the studies have used a cross-sectional approach mainly through OLS regressions to examine the relationship. Hence, it would be worth conducting further investigation based on longitudinal data that adopt different methodological approaches such as panel data (i.e. cross-sectional time series data) with the related beneficial consequences. For instance, through panel data analysis not only variations between firms but also within firms (and so their behaviour across time) could be accounted for. Furthermore, a panel data approach would control for endogeneity issues between variables and unobserved heterogeneity. Employing a fixed-effects estimator (if appropriate) would further help with unobserved factors such organisational culture, age of the hospital facilities and so forth. Panel data is also suitable for including variables at different levels of analysis (for instance individual, organisational and system levels). Essentially, the results of the empirical analysis would be more robust and generalizable.

Apart from methodological concerns, it appears that the large majority of the studies have focused on the Anglo-American context, with only a handful of papers based on other European health systems. Without denying the importance of lessons drawn from the UK and the US health systems, there seems to be a clear need to expand research to other countries. In this way, it would be possible to analyse the impact of country-specific governance models. For instance, in France the hospital governance structure is dramatically different from the Anglo-American model with a far greater presence of clinicians on advisory boards. However, there is no denying that the hospital corporatisation process has led to a widespread adoption of the governing board model typical of the Anglo-American context in countries such as Belgium, the Netherlands, Portugal, Spain, and so forth [5]. Similarly, it would be interested to draw comparisons between health systems that follow either the Beveridge or the Bismarkian model. Additionally, we would welcome more ambitious studies that directly take a multi-country approach. So far, only Dorgan et al. [13] have ventured down this line of research but their study is limited in scope (mainly focused on investigating the impact of management systems) and methodologically weak. As far as we are aware, there is also a dearth of research from countries outside North America and Europe. This line of research could usefully expand the emerging evidence on the importance of hospital governing board oversight on safety and quality concerns and the role played by clinicians in this respect [24, 51].

A further line of useful (if not needed) research could be developed by focusing at the middle and junior management levels. The role played by clinicians at the strategic decision making level is obviously crucial, as shown by the studies analysed in this review, however more needs to be understood on the type of impact clinical leadership has at the operational level. The large majority of hospitals (at least in the developed world) have adopted the John Hopkins Hospital clinical directorate model, where a clinician is given managerial responsibilities in relation to human and financial resources and is held accountable for the performance of the directorate. The extensive adoption of this organisational model lends itself to a comparative research design, especially considering that there is already qualitative evidence suggests that the implementation of clinical directorates has led to different interpretations and practices across countries [52].

Finally, quantitative approaches can show the existence of a positive relationship (and the direction of influence) between clinical leadership and hospital performance, but they cannot explain the why and how. So far, most of the explanations provided (summarised in Fig. 3) are essentially based on educated assumptions derived from a handful of qualitative studies. Expertise at the individual and clinical levels is undoubtedly crucial, but it still far from clear how far other potential explanatory factors relating to, for instance, the leadership style of clinicians, the hospital cultural context, and directors’ collective behaviour within boards are influencing the impact of clinical leadership [53]. So, we would also encourage further investigation based on in-depth qualitative approaches, perhaps by observing hospital board meetings or by interviewing/surveying hospital senior leaders.

Besides the identifications of research gaps and the suggestion for future studies, a reference to the theoretical implications of the findings reported by our review is worthy of note. While more work is indeed needed, we can conclude that our explanatory model supports the reasoning underpinning clinical leadership and is in line with the policy makers’ effort to support the development of clinicians in leadership position. There is also growing evidence suggesting that clinicians are increasingly seeing in positive terms their involvement in managerial positions, and this hybrid role is becoming engrained in their understating of professional responsibilities and duties [54, 55]. This is linked to what Noordegraaf [56] defines as ‘organizing professionalism’, essentially a new form of hybrid professionalism. This categorisation reflects the emergence of clinical professionals who are no longer solely engaged with providing care to patients, but they also are involved in the organizing of the healthcare organization they are working for. In this sense, *organizing* becomes an embedded component of professional work centring around activities such as the arrangement of inter-professional collaboration, the design and implementation of innovative projects, the management of scarce resources, the alignment of decision processes in accordance with financial constraints, and the management of relationship with multiple stakeholders.

### Declarations

This publication is supported by COST. This article has been published as part of BMC Health Services Research Volume 16 Supplement 2, 2016: Medicine and management in European public hospitals. The full contents of the supplement are available online at http://bmchealthservres.biomedcentral.com/articles/supplements/volume-16-supplement-2.

### Ethics approval and consent to participate

Not applicable.

### Consent for publication

Not applicable.

## Abbreviations

BoD: Board of Directors
CEO: Chief Executive Officer
MD: Medical Director
NHS: National Health Service
